# Thomas precession, relativistic torque, and non-planar orbits

**DOI:** 10.1140/epjc/s10052-025-13747-4

**Published:** 2025-01-21

**Authors:** Andrzej Czarnecki, Andrei Zelnikov

**Affiliations:** Department of Physics, University of Alberta, Edmonton, AB T6G 2E1 Canada

## Abstract

We analyze the angular momentum balance for a particle undergoing Thomas precession. The relationships among relativistic torque, the center of mass, and the center of inertia for a spinning particle are clarified. We show that spin precession is accompanied by orbital angular momentum precession, and present examples of the resulting out-of-plane motion.

## Introduction

In classical physics, spin is the intrinsic angular momentum of a rotating body, such as a gyroscope. The spin orientation remains constant in the absence of external forces, but can change under acceleration. This relativistic effect, known as Thomas precession [1], was discovered by Föppl and Daniell, [2], and independently by Silberstein [3]; for further historical context, see [4].

Acceleration also induces radiation. When the spin precesses, does the orbital angular momentum $\varvec{L}$ adjust to conserve the total angular momentum $\varvec{J}$, or is the imbalance radiated away? One might also consider a periodic exchange of angular momentum between the particle and its near-zone electromagnetic field.

Here we find that when a charged spinning particle orbits a nucleus, $\varvec{L}$ changes as the spin precesses, causing the particle to move in and out of its average orbital plane.

We consider the limit of the particle’s classical radius much smaller than the curvature radius of its worldline [5]. Both radiation and periodic exchange are then negligible in comparison with the change of $\varvec{L}$.

We characterize the resulting out-of-plane motion. This relativistic effect requires a consistent description of the orbiting particle. The spin is typically defined in the frame comoving with the particle, while precession and radiation are more easily described in the frame centered at the nucleus at rest (Lab). This dual approach has often led to confusion. We clarify and visualize the relativistic dynamics of the spinning particle from the perspective of a Lab observer.

Precession of a gyroscope requires torque. However, a force acting on the gyroscope’s center of mass exerts no torque in its rest frame. Muller [6] resolved this apparent paradox, and Rębilas [7] further refined the solution, showing that while there is no torque in the rest frame, the force accelerating the gyroscope exerts a torque in the Lab frame.

To understand the origin of this torque, it is important to distinguish between the center of mass (CM) and the center of inertia (CI) [6]. A consistent treatment of Thomas precession requires relativistic generalizations of spin, torque, CM, and CI. Despite the long history and extensive literature on this topic (for recent work and further references see [8, 9]), we believe that the relativistic description of these quantities still lacks necessary clarity. In this paper, we outline the peculiarities of the relativistic motion of a spinning particle.

We assume the nucleus is pointlike and sufficiently massive that its motion and magnetic moment are negligible. The central electric force it exerts on the particle is the only force we consider, which we refer to as the force. We assume that the electric field does not probe the particle’s internal structure.

We refer to an instantaneous inertial frame comoving with the particle as the Rest frame and mark with a bar quantities defined in that frame. The Lab frame time is denoted by *t*, and the particle’s proper time by $\tau$.

Section 2 clarifies the differences between CM and CI. In Sect. 3, we introduce the kinematics and derive the evolution of the spin with respect to $\tau$, known as Fermi transport. Section 4 discusses relativistic angular momentum and spin. In Sect. 5, we present examples of spin precession and illustrate its dynamics with plots. Additional examples and technical details related to the dynamics of CM and CI are provided in Appendices A and B.

We use the metric signature $(-,+,+,+)$ and adopt units with the speed of light $c = 1$. Summation is implied over repeated indices. Greek letters ($\alpha , \beta , \dots = 0,1,2,3$) denote spacetime components, while Latin letters ($i, j, \dots = 1,2,3$) denote spatial components. We work in Cartesian coordinates, and for the Levi–Civita symbol, we adopt the sign convention $\varepsilon _{0123} = 1 = -\varepsilon ^{0123}$, with $\varepsilon _{123} = \varepsilon ^{123} = 1$ [10].

## Centers of inertia and of mass

In this section, we carefully define the centers of inertia and mass and provide an intuitive explanation of their differences.

The particle’s inertial properties are described by the stress-energy tensor $T^{\alpha \beta }$, obtained by varying the classical action with respect to the metric. This tensor is symmetric, $T^{\alpha \beta } = T^{\beta \alpha }$ [11]. We use it to study the particle’s dynamics in the Lab frame. The energy of the system is the time component $P^0$ of the momentum four-vector $P^\alpha$,

2.1 $$\begin{aligned} P^{0}(t)=\int {\mathrm d}^3x \,T^{0 0}(x). \end{aligned}$$

The integral is computed over a hypersurface of events that are simultaneous in the Lab frame, with $t = {\textrm{const}}$.

The position of the particle’s center of inertia (CI; also called “Lab frame centroid” [12]) in the Lab frame,

2.2 $$\begin{aligned} R^{\alpha }_{\tiny {\text{ CI }}}(t)={1\over P^0} \int {\mathrm d}^3x \,x^{\alpha } T^{0 0}(x), \end{aligned}$$

corresponds to our intuition about where the total energy of the system is centered. The numerator in Eq. (2.2) is the dipole moment of the energy of the system. Concentrating the whole energy of the system in its CI does not change its energy dipole moment [13].

Unlike CI, the position of the CM,

2.3 $$\begin{aligned} \bar{R}^{\alpha }= \left( \bar{t}, {\int {\mathrm d}^3\bar{x}~\bar{x}^i\bar{T}^{0 0}(\bar{x})\over \int {\mathrm d}^3\bar{x}~\bar{T}^{0 0}(\bar{x})} \right) , \end{aligned}$$

is not Lorentz covariant: it depends on the hypersurface $\bar{t}={\textrm{const}}$ in the integrals. Although CM and CI coincide in the Rest frame, they may differ in the Lab frame.

The difference between CI and CM can be understood with the example of a bicycle wheel [6]. In the frame of a street, the CI of a moving wheel is above the hub because the speeds of the spokes are larger there than below the hub (the point of the wheel-street contact is at rest with respect to the street, while the top of the wheel is moving with twice the speed of the bike). Elements of the wheel above the hub have more kinetic energy.

To explain the interplay of the dynamics of the particle’s spin and its orbital motion, we define the part of the total angular momentum which describes the intrinsic rotation of the particle. The orbit is the trajectory of the CM. It is moving with the four-velocity $U^{\alpha }=P^{\alpha }/M$ [13], where $M=\sqrt{-P^{\alpha }P_{\alpha }}$ is the particle’s mass. This definition formalizes the intuition that the particle is at rest in the CM rest frame and the Lorentz invariant energy $\bar{P}^{0}=-P^{\alpha }U_{\alpha }$ equals the invariant mass *M*. From now on we use this choice of $U^{\alpha }$ to describe the CM velocity.

For an isolated system the total stress-energy tensor is conserved, $\partial _\lambda T^{\alpha \lambda }=0$, hence, in the absence of radiation to infinity, momentum $P^{\alpha }$ is constant. If an external force, such as an external Maxwell field, acts on the particle, then $\partial _\lambda T^{\alpha \lambda }=f^{\alpha },$ where the four-vector $f^{\alpha }(x)$ is the density of the external force.

## Fermi transport

Fermi transport will be a key tool in our discussion of the orbital motion of a spinning particle. The crucial result of this section is Eq. (3.8), which describes the evolution of the spin with respect to proper time.

In classical physics, we consider the spin as a three-vector $\textbf{S}$. In special relativity, a vector that is purely spatial in the Rest frame acquires a time component in the Lab frame. Thus, we treat the spin as a four-vector.

In the Lab frame, the particle’s worldline has coordinates $R^{\alpha }(t)=\big (R^0(t),R^{i}(t)\big )$ with $R^0(t)=t$. The four-velocity $U^{\alpha }$ of the particle is $U^{\alpha }={d R^{\alpha }\over d\tau }$. The worldline of a massive particle is timelike,

3.1 $$\begin{aligned} U^{\alpha }U_{\alpha }=-1. \end{aligned}$$

In the Rest frame, $\bar{U}^{\alpha } = (1, 0, 0, 0)$. The fact that the spin four-vector $S^{\alpha }$ reduces to a spatial vector in the Rest frame can be invariantly expressed through orthogonality,

3.2 $$\begin{aligned} S^{\alpha }U_{\alpha }=0. \end{aligned}$$

Spin’s magnitude is constant,

3.3 $$\begin{aligned} S^{\alpha }S_{\alpha }={\textrm{const}}. \end{aligned}$$

In the Lab frame the four-velocity reads $U=\gamma (1,\varvec{V})$ with $\varvec{V}={d \varvec{R}/dt}$ and $\gamma =1/\sqrt{1-\varvec{V}^2}$. Because of (3.1), four-acceleration, $w^{\alpha }={d U^{\alpha }\over d\tau }$, is orthogonal to the four-velocity

3.4 $$\begin{aligned} w^{\alpha }U_{\alpha }=0. \end{aligned}$$

Orthogonality (3.2) and constant magnitude (3.3) have to be satisfied at any point of the worldline,

3.5 $$\begin{aligned} {d\over d\tau }(S^{\alpha }U_{\alpha })=0 \quad {d\over d\tau }(S^{\alpha }S_{\alpha })=0. \end{aligned}$$

The four-dimensional rotation, or infinitesimal Lorentz transformation, is defined by an antisymmetric matrix,

3.6 $$\begin{aligned} {dS^{\alpha }\over d\tau }=\Omega ^{\alpha \beta }S_{\beta } \quad \Omega ^{\alpha \beta }=-\Omega ^{\beta \alpha }. \end{aligned}$$

$\Omega ^{\alpha \beta }S_{\alpha }S_{\beta }=0$ ensures the second condition (3.5).

The matrix $\Omega ^{\alpha \beta }$ has non-zero components only in the plane of rotation. It is useful to think about rotation in a plane (it can be defined in arbitrary dimensions greater than one) rather than around an axis which can be defined only in three dimensions [10].

The rotation plane is defined by the four-velocity $U^{\alpha }$ and the four-acceleration $w^{\alpha }$. The only antisymmetric structure defined by these vectors has the form [10]

3.7 $$\begin{aligned} \Omega ^{\alpha \beta }=a(U^{\alpha }w^{\beta }-w^{\alpha }U^{\beta }). \end{aligned}$$

$a=1$ follows from the first condition in (3.5). Taking into account conditions (3.2) and (3.4), we obtain

3.8 $$\begin{aligned} {dS^{\alpha }\over d\tau }=U^{\alpha }w^{\beta }S_{\beta }. \end{aligned}$$

This equation defines a transport law of the spin vector along an arbitrary worldline. Proposed by Fermi [14], it is known as the Fermi transport. It leads to the Bargmann–Michel–Telegdi (BMT) equation [15] for the dynamics of the spin (see [16] for a simple derivation).

## Orbital and spin angular momenta

We now derive the crucial Eq. (4.9) for the difference of positions of the centers of inertia and of mass.

Consider the orbital angular momentum $L^{\alpha }$, the spin $S^{\alpha }$, and the total angular momentum $J^{\alpha }=L^{\alpha }+S^{\alpha }$. Instead of these vectors it is useful to introduce their dual antisymmetric tensors $J^{\alpha \beta }$, $L^{\alpha \beta }$, and $S^{\alpha \beta }$, defined by (similarly for *J* and *L*)

4.1 $$\begin{aligned} S_{\alpha }=-\frac{1}{2} \varepsilon _{\alpha \rho \mu \nu }U^{\rho }S^{\mu \nu }, \quad S^{\mu \nu }=\varepsilon ^{\mu \nu \alpha \beta }U_{\alpha }S_{\beta }. \end{aligned}$$

$J^{\alpha \beta }$ is given (Ref. [10], §5.11) by the integral over a spacelike surface $t={\textrm{const}}$ in Lab,

4.2 $$\begin{aligned} J^{\alpha \beta }=\int {\mathrm d}^3x \,\Big [ x^{\alpha }T^{0\beta }-x^{\beta }T^{0\alpha }\Big ], \end{aligned}$$

The force being central, $J^{\alpha \beta }$ is conserved, provided that we neglect back-reaction effects of radiation to infinity. The orbital angular momentum is

4.3 $$\begin{aligned} \begin{aligned} L^{\alpha \beta }(t)&=\int {\mathrm d}^3x \,\Big [ R^{\alpha }(t)T^{0\beta }-R^{\beta }(t)T^{0\alpha }\Big ]\\&=R^{\alpha }(t)P^{\beta }-R^{\beta }(t)P^{\alpha }, \end{aligned}\end{aligned}$$

where $R^{\alpha }(t)$ is the worldline of CM. Both orbital $L^{\alpha \beta }(t)$ and intrinsic $S^{\alpha \beta }(t)$ angular momenta depend on *t*.

Similarly to (3.2) the spin tensor is orthogonal to the four-velocity (the supplementary spin condition [17]),

4.4 $$\begin{aligned} S^{\alpha \beta }U_{\beta }=0, \end{aligned}$$

Fermi transport (3.8) for the spin tensor is

4.5 $$\begin{aligned} {dS^{\alpha \beta }\over d\tau }=-\big (U^{\alpha }S^{\beta \lambda }-U^{\beta }S^{\alpha \lambda }\big ) w_{\lambda }. \end{aligned}$$

Using definitions (4.2), (4.3) one writes

4.6 $$\begin{aligned} S^{\alpha \beta }(t)=\int {\mathrm d}^3x \,\Big [ \big (x^{\alpha }-R^{\alpha }(t)\big )T^{0\beta }-\big (x^{\beta } -R^{\beta }(t)\big )T^{0\alpha } \Big ]. \end{aligned}$$

In Lab, $R^{\alpha }(t)=x^0=t$, and $S^{\alpha 0}$ becomes

4.7 $$\begin{aligned} S^{\alpha 0}(t)=\int {\mathrm d}^3x \, \big (x^{\alpha }-R^{\alpha }(t)\big )T^{00}, \end{aligned}$$

the energy dipole moment about CM. Using the CI definition (2.2) and the total energy (2.1),

4.8 $$\begin{aligned} S^{\alpha 0}(t)=P^0\big [ R^{\alpha }_{\tiny {\text{ CI }}}(t)-R^{\alpha }(t)\big ]. \end{aligned}$$

The shift of the CI relative to the CM is determined by the energy and the $S^{\alpha 0}$ spin tensor component,

4.9 $$\begin{aligned} \Delta R^{\alpha }(t)=R^{\alpha }_{\tiny {\text{ CI }}}(t)-R^{\alpha }(t)={S^{\alpha 0}(t)\over P^0}. \end{aligned}$$

This shift of the CI is zero in the Rest frame but not in the Lab frame.

## Relativistic spin precession

**Fig. 1:**
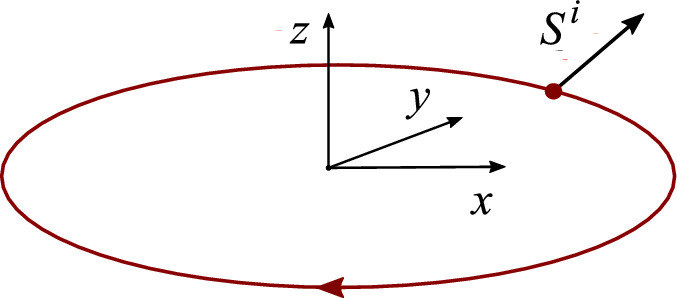
The worldline $R^i(t)$ of the particle with the spin $S^i$

We consider a particle moving along a given trajectory. For simplicity we choose the motion of CM to be a circle and study the dynamics of the spin and its relation to $\Delta R^{\alpha }$ (Fig. 1). The back-reaction of the spin on the motion of CM is considered in Appendix B.

Let *r* be a radius of the orbit and $\omega$ the frequency of rotation along the orbit. Then

5.1 $$\begin{aligned} R^{\alpha }=(t,R^x,R^y,R^z)=(t,r\sin \omega t,r\cos \omega t, 0). \end{aligned}$$

The four-velocity and four-acceleration read

5.2 $$\begin{aligned} U^{\alpha }&={dR^\alpha \over d\tau }=\gamma ~ (1, V^x,V^y,V^z) \end{aligned}$$

5.3 $$\begin{aligned}&=\gamma ~ (1, V\,\cos \omega t, -V\,\sin \omega t,0), \end{aligned}$$

5.4 $$\begin{aligned} w^\alpha&={dU^\alpha \over d\tau } =-\gamma ^2 \omega ^2 r ~[0,\, \sin \omega t,\,\cos \omega t,\,0]. \end{aligned}$$

Fermi transport (3.8) leads to $\partial _t S^z=0$ and

5.5 $$\begin{aligned} \begin{aligned}&\partial _t S^0=-\gamma ^2\omega ^2 r\,(\sin \omega t ~S^x+\cos \omega t~S^y),\\&\partial _t S^x=-\gamma ^2\omega ^3 r^2 \cos \omega t~(\sin \omega t ~S^x+\cos \omega t~S^y),\\&\partial _t S^y=+\gamma ^2\omega ^3 r^2 \sin \omega t~(\sin \omega t ~S^x+\cos \omega t~S^y). \end{aligned}\nonumber \\ \end{aligned}$$

The solution (p. 175 in Ref. [10]) is $S^z={\textrm{const}}$ and

5.6 $$\begin{aligned} S^0&=\tilde{S}\,\sqrt{\gamma ^2-1}\cos \gamma \omega t,\nonumber \\ S^x&=\tilde{S}\Bigg ({\gamma +1\over 2}\cos [(\gamma -1)\omega t]+{\gamma -1\over 2}\cos [(\gamma +1)\omega t]\Bigg ),\nonumber \\ S^y&=\tilde{S}\Bigg ({\gamma +1\over 2}\sin [(\gamma -1)\omega t]-{\gamma -1\over 2}\sin [(\gamma +1)\omega t]\Bigg ). \end{aligned}$$

The Thomas precession rate $\Omega _T$ can be read off the first terms in (5.6), $\Omega _T=(\gamma -1)\omega .$ The direction of rotation is opposite to the rotation of the particle, as can be deduced from comparison with the direction of rotation of the velocity vector $V^{x,y}=V (\cos \omega t,- \sin \omega t)$. The second terms in Eqs. (5.6) describe oscillations with frequency $(\gamma +1)\omega$. Their magnitude, proportional to $(\gamma -1)$, is small in the non-relativistic case when $\gamma -1\rightarrow V^2/2\ll 1$.

Equation (4.1) provides (0*i*) components of the spin tensor,

5.7 $$\begin{aligned}&\left[ S^{0x}, S^{0y}, S^{0z} \right] = \sqrt{\gamma ^2-1} \nonumber \\  &\qquad \cdot \left[ S^z \sin \omega t, S^z \cos \omega t, -\tilde{S}\sin \gamma \omega t \right] . \end{aligned}$$

Substituted into (4.9), these solutions give the CI shift in terms of the invariant mass $M=P^0/\gamma$,

5.8 $$\begin{aligned}&\left[ \Delta R^{x}(t), \Delta R^y(t), \Delta R^z(t) \right] ={ V\over M} \nonumber \\  &\quad \cdot \left[ -S^z \sin \omega t, -S^z \cos \omega t, \tilde{S} \sin \gamma \omega t \right] . \end{aligned}$$

This result is valid for any value of $\gamma$. Definition (5.8) of $\Delta R^{\alpha }$ differs from Eqs. (16–18) in Ref. [7] by a factor $\gamma$, inconsequential for their non-relativistic analysis.

Equations (5.8) show that the dynamics of CI is a superposition of two motions with frequencies $\omega$ and $\gamma \omega$. Thomas precession frequency $\Omega _T$ equals their difference while the frequency of the oscillations is their sum. In the plane of the orbit CI is moving synchronously with the position of the particle. Projection of the CI orbit on the *xy* plane moves in a circle with radius $R_{\tiny {\text{ CI }}}=r - S^z V/ M$ with frequency $\omega$. Depending on the sign of $S^z$ this circle can be either inside or outside the CM orbit. $\Delta R^{z}$ describes the shift of CI above and below the orbital plane with frequency $\gamma \omega$. For an inertial motion the relativistic shift of the CI was defined in Eq. (48) of Ref. [13].

**Fig. 2:**
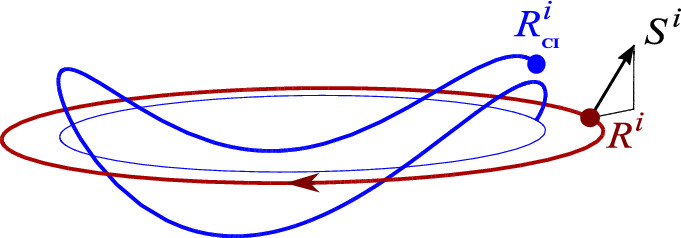
Typical trajectory of the CI (blue curve), when the particle moves in a circle. Here the spin’s tilt angle is $\pi /4$, ${\bar{S}/ M}=0.3$, and $\gamma =2.3$. Projection of the CI trajectory onto the orbital plane is the thin (blue) circle with radius $r-VS^z/M$

**Fig. 3:**
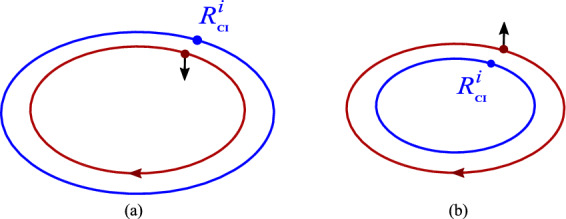
**a** When the spin is parallel to the orbital angular momentum $L^i$, the CI orbit has a larger radius than that of CM. **b** If the spin is antiparallel to $L^i$, CI is moving in a circle with a smaller radius

**Fig. 4:**
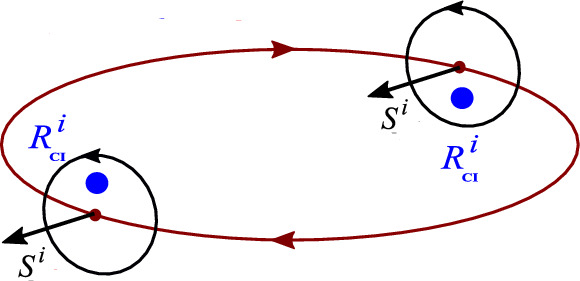
When the spin lies in the orbital plane, the CI shifts in the *z* direction above or below the orbital plane depending on the orientation of the spin with respect to the velocity

**Fig. 5:**
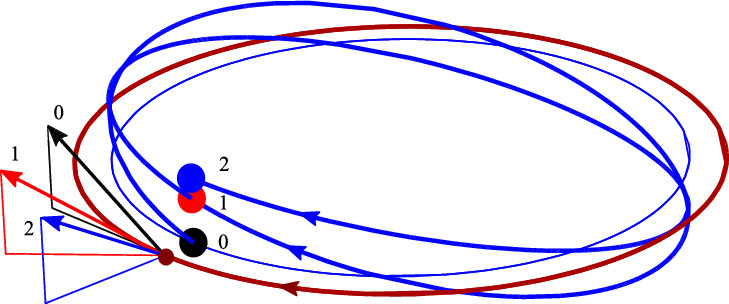
For small velocities the CI orbit is approximately a plane slightly tilted relative to the CM orbit and its orientation precesses with the Thomas frequency. Three spin vectors represent orientations of the spin at $t=0$ and after two turns around the CM orbit ($t=2,4\pi /\omega$). Thin (blue) circle is the projection of the CI trajectory onto the orbital plane

**Fig. 6:**
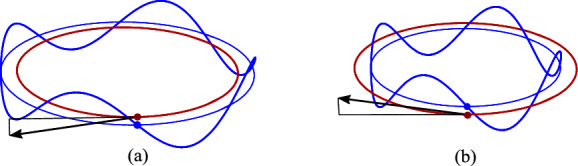
When $\gamma$ is rational, the CI trajectory is closed. Here two orientations of the spin are shown for $\gamma =5$

Figures 2, 3, 4, 5 and 6 show CI trajectories for various spin magnitudes, orientations, and $\gamma$ factors. For comparable spin and orbital angular momenta, CI can move even along a line orthogonal to the CM orbital plane.

A generic trajectory is illustrated in Fig. 2. Here the orbital angular momentum is pointing downwards (negative) while the spin has a positive $S^z$ component. This is why CI moves inside the circular CM orbit. The projection of the CI trajectory is the thin (blue) line. It is a circle. Because the spin also has non-vanishing components in the orbital plane, the CI trajectory wiggles above and below the orbital plane with frequency $\gamma \omega$.

If the spin $S^i$ is orthogonal to the orbital plane, CI moves along the circle inside or outside the CM orbit, depending on the sign of the $S^z$ component, see Fig. 3.

The direction of the CI shift can be understood with a model of an extended rotating body. For parallel spin and orbital angular momenta, $\Delta R^{i}(t)$ is in the orbital plane. In this case the velocities of the orbital motion of the CM and of the outer parts of the rotating body add up, while in the inner part they partially cancel. Therefore the relativistic ${\gamma }$ factor is larger in the outer parts. The shift of the CI is the difference $x^{\alpha }-R^{\alpha }(t)$ averaged with the weight $T^{00}(x)/P^{0}$. The ${\gamma }$ factor in $T^{00}$ gives outer parts a larger weight and the CI shifts radially outwards.

For the spin antiparallel to the orbital angular momentum, the same logic dictates that the CI shifts radially inwards. In both cases the CI trajectory is circular. If the spin is considerably larger than the orbital momentum, $\Delta R^{i}$ can exceed the radius of the orbit. For an antiparallel spin, the shift overshoots the orbital center and the CI is on the opposite side of the nucleus. The direction and the frequency of rotation coincide with those of the orbital motion. For a large antiparallel spin the CI can shift even beyond the CM orbit.

If the spin $S^i$ lies in the orbital plane, the CI shift is orthogonal to the plane (see Fig. 4). The CI trajectory lies on a cylinder of the same radius as the CM orbit. It oscillates above and below the orbital plane with the frequency $\gamma \omega$, with $\gamma$ accounting for the Thomas precession. Without precession the spin would always point in the same direction and the frequency of oscillations about the orbital plane would be $\omega$. The difference of oscillation frequencies of $R^{x,y}_{\tiny {\text{ CI }}}$ and $R^{z}_{\tiny {\text{ CI }}}$ components is the Thomas frequency $(\gamma -1)\omega$ as measured in the Lab frame.

The direction of the CI shift is shown in Fig. 4. If the upper parts of the spinning matter move in the same direction as the orbital motion, the shift is up. Otherwise it is down. When the spin is parallel to the orbital velocity, relativistic factors above and below the plane are the same and the shift vanishes. Detailed computations of CI for this gyroscope are shown in Appendix A.

For a generic orientation of the spin, the CI motion is a linear combination of the previous two cases. The trajectory lies on a cylinder with the radius determined by the *z* component of the spin, as in the case of an (anti)parallel spin. Seen from the pole, the trajectory is circular. From an arbitrary observation point, Fig. 2, we see a linear combination of an orbital motion with the frequency $\omega$ and oscillatory motions above and below the equatorial plane with frequency $\gamma \omega$.

For a non-relativistic orbital motion, $\gamma -1\simeq V^2/2\ll 1$, CI trajectory resembles a circle in a slightly tilted plane whose orientation slowly evolves with the Thomas frequency $\omega V^2/2$. Spin rotates, as depicted in Fig 5.

**Fig. 7:**
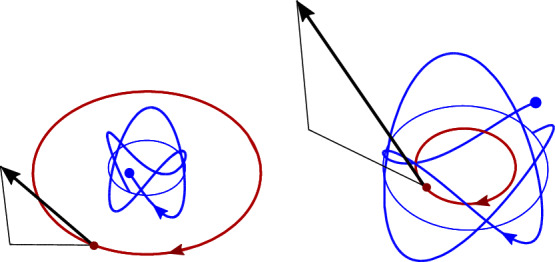
If the spin of the particle has a large *z* component of opposite sign than the orbital momentum, the CI can shift close to the orbital center. For larger spins CI may shift to the other side of the orbit

Figures 6 and 7 present the CI motion for various orientations of the spin and velocity. If $\gamma$ is rational, out of plane oscillations and the orbital rotation synchronize and CI moves along a closed curve, see Fig. 6.

In Fig. 7 magnitudes of the spin and the orbital momentum are comparable. Remarkably, the CI can be on the same side of the nucleus as CM or, for very large spins, on the other side. As its radius shrinks, the cylinder on which the CI trajectory is winding can degenerate to a line along which the CI oscillates with frequency $\gamma \omega$.

As we have seen, the distance between CM and CI can evolve in a sophisticated way. Spin precesses but the total angular momentum remains constant. Naturally, spin should affect the orbital angular momentum and, as a result, the motion of CM. The dynamics of the linear momentum of a spinning charged particle was solved by Frenkel [18]. Contribution of radiation was derived later [5]. Appendix B describes this interesting effect.

## Discussion

We have described Thomas precession of a spinning point particle, assuming that its center of mass (CM) moves along a given trajectory and that energy is conserved. At any moment, this trajectory can be considered as a segment of a circular orbit. Since our analysis is local in time, our conclusions apply not only to circular orbits but also to more complicated trajectories, provided that energy is conserved.

However, if the accelerated particle is charged, it radiates energy. Fortunately, Thomas precession appears in the leading (first) order of acceleration, while radiation effects are of the second order in both acceleration and charge *q*. The effect of radiation on Thomas precession is negligible, provided that the self-interaction terms (which are proportional to $q^2$ and hence to the square of the acceleration) in the equation of motion are small compared to the external force (which is proportional to *q*). This assumption applies to a point particle, whose structure is not influenced by the external field, and where the radius of curvature of the worldline is large compared to the classical radius $\sim q^2/m$. This argument accounts for the electromagnetic energy of the charge and applies equally to spinless particles. Similarly, periodic exchange of angular momentum with the particle’s near-zone electromagnetic field is a higher-order effect in *q* and thus negligible in our approximation.

In the case of spinning particles, another effect arises: the back reaction of the spin on the center of mass (CM) orbit of the particle. This effect is described by the Frenkel equation (B1) [18, 19], as detailed in Appendix B.

The back-reaction correction to the orbit is also of the second order in acceleration. Thus, similar to the radiation effects, it is small in the point particle approximation when the spin is smaller than the orbital angular momentum.

In Fig. 7, we chose large spin values only for visualization purposes. Corrections to the point particle orbit due to spin and radiation-related self-interaction should be considered perturbatively.

Due to the spin-orbit interaction, the motion of a spinning particle can be quite complicated. Such complex behavior has also been observed in gravitational physics. The motion of a spinning particle in the gravitational field of a black hole is described by the Mathisson–Papapetrou–Dixon equations [20–23], which serve as the gravitational analogue of Eq. (4.5). For large spins, the spin-orbit interaction can even lead to chaotic motion out of the orbital plane [24]. This chaotic regime may significantly affect the power and shape of gravitational radiation from colliding black holes. It would be interesting to explore similar effects for the electromagnetic interaction of spinning particles.

## Figures and Tables

**Fig. 8:**
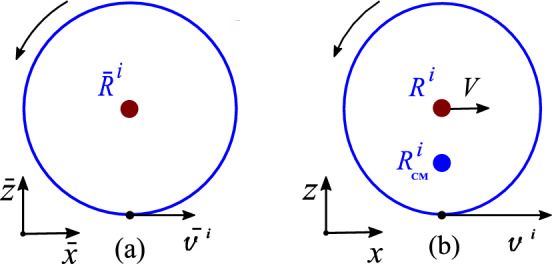
Panel **a** represents the rotating ring in its CM frame. In the Lab frame **b** it moves with the velocity *V*. Speeds and thus energies of the upper parts are smaller than of the lower ones, so the CI shifts below the center of the ring

## Data Availability

My manuscript has no associated data. [Author’s’ comment: Data sharing not applicable to this article as no datasets were generated or analysed during the current study.]

## Appendix A: Spinning ring in the Lab frame

As an illustration of physics behind the relativistic shift of the CI relative to the CM we consider a simple model of a gyroscope as a rotating ring proposed by Muller [6], shown in Fig. 8.

Let $\rho$ be the radius of the ring and $\mu$ the linear mass density. In the center of mass frame every element of the ring, characterized by angle $0\le \psi <2\pi$, moves around a circle in the $x-z$ plane,

A1 $$\begin{aligned} {[}\bar{x}(\bar{t}), \bar{y}(\bar{t}), \bar{z}(\bar{t})] =\rho [\sin (\Omega \bar{t}+\psi ), 0, -\cos (\Omega \bar{t}+\psi )], \end{aligned}$$

with the four-velocity

A2 $$\begin{aligned} \begin{aligned}&\bar{u}^{\mu }=\bar{\gamma }~ [1, \bar{v}\cos (\Omega \bar{t}+\psi ),0, \bar{v}\sin (\Omega \bar{t}+\psi )],\\&\bar{v}=\Omega \rho \quad \bar{\gamma }={1\over \sqrt{1-\bar{v}^2}}. \end{aligned}\end{aligned}$$

Here $\bar{x}^{\alpha }$ are coordinates in the CM frame, $\Omega$ is the angular velocity of the ring, and its spin tensor is

A3 $$\begin{aligned} \bar{S}^{\mu \nu }=\int _0^{2\pi } (\mu \rho d\psi ) [\bar{x}^{\mu }\bar{u}^{\nu }-\bar{x}^{\nu }\bar{u}^{\mu }] \end{aligned}$$

With $\int _0^{2\pi } d\psi \, \sin ,\cos (\Omega \bar{t}+\psi )=0$, we obtain

A4 $$\begin{aligned} \begin{aligned}&\bar{S}^{xz}=2\pi \mu \bar{\gamma } \rho ^2 \bar{v}=\bar{E}\Omega \rho ^2\equiv \bar{S}, \\&\bar{S}^{0x}=\bar{S}^{0y}=\bar{S}^{0z}=\bar{S}^{xy}=\bar{S}^{yz}=0. \end{aligned}\end{aligned}$$

Invariant mass $M=\sqrt{-P^{\alpha }P_{\alpha }}$ of the ring is

A5 $$\begin{aligned} M=\bar{E}=2\pi \mu \rho \bar{\gamma }. \end{aligned}$$

Consider this rotating ring in the Lab frame. Lorentz transformation to the Lab coordinates $x^{\alpha }$ is (assuming that CM moves with velocity *V* along *x*)

A6 $$\begin{aligned} \begin{aligned}&t=\gamma _{\tiny {\text{ V }}} (\bar{t}+V\bar{x}) \quad \gamma _{\tiny {\text{ V }}}={1/ \sqrt{1-V^2}},\\&x=\gamma _{\tiny {\text{ V }}} (\bar{x}+V\bar{t}) \quad y=\bar{y}\quad z=\bar{z}. \end{aligned}\end{aligned}$$

Velocity $v^{i}=dx^i/dt$ of a ring element transforms as

A7 $$\begin{aligned} v^x={\bar{v}^x+V\over 1+V\bar{v}^x}\quad v^{y,z}={\bar{v}^{y,z} \over \gamma _{\tiny {\text{ V }}} (1+V\bar{v}^x)}. \end{aligned}$$

The Lab four-velocity of a ring element reads

A8 $$\begin{aligned} u^{\mu }={\gamma }~ [1, v^x,v^y,v^z]\quad \gamma =1/\sqrt{1-\varvec{v}^2}. \end{aligned}$$

(A7) and (A2) give $\gamma =\bar{\gamma }\gamma _{\tiny {\text{ V }}}\,(1+V\bar{v}^x)$. Therefore

A9 $$\begin{aligned} u^{\mu } = \bar{\gamma }[\gamma _{\tiny {\text{ V }}}\,(1+V\bar{v}^x), \gamma _{\tiny {\text{ V }}}\,(\bar{v}^x+V), \bar{v}^y, \bar{v}^z ]. \end{aligned}$$

Now compute the component $S^{\alpha 0}$ (4.7) of the spin tensor, defining the CM shift. The stress-energy tensor is localized on the ring so the spatial integration reduces to the integration over the angle $\psi$. We obtain

A10 $$\begin{aligned} S^{\alpha 0}=\int \limits _0^{2\pi } (\mu \rho d\psi ) (x^{\alpha }-R^{\alpha })u^{0}. \end{aligned}$$

Substitution of (A9) and (A2) leads to $S^{y 0}=0$ and

A11 $$\begin{aligned} \begin{aligned}&S^{x 0}=\mu \rho ^2\bar{\gamma }\gamma _{\tiny {\text{ V }}}^2\int \limits _0^{2\pi }d\psi \, \sin (\Omega \bar{t}+\psi )[1+V\bar{v}\cos (\Omega \bar{t}+\psi )] ,\\&S^{z 0}=-\mu \rho ^2\bar{\gamma }\gamma _{\tiny {\text{ V }}}\int \limits _0^{2\pi }d\psi \, \cos (\Omega \bar{t}+\psi )[1+V\bar{v}\cos (\Omega \bar{t}+\psi )]. \end{aligned}\end{aligned}$$

All quantities should be evaluated in the Lab frame, at constant *t* rather than $\bar{t}$. Following Muller [6], we express them in terms of the Lab time *t*. This is important because the CM shift results from two equal [6] contributions: i) Faster moving parts of the ring are heavier; ii) Time delay depends on the part of the ring as seen in the Lab. Using $t=\gamma _{\tiny {\text{ V }}} (\bar{t}+V\bar{x}),$

A12 $$\begin{aligned} \cos ,\sin (\Omega \bar{t}+\psi )=\cos ,\sin \left( {\Omega \over \gamma _{\tiny {\text{ v }}}} t+\psi -V\Omega \bar{x}\right) . \end{aligned}$$

We use $\psi + \Omega t /\gamma _{\tiny {\text{ v }}} \equiv \varphi$ as the coordinate on the ring; at $t={\textrm{const}}$ it differs from $\psi$ by a constant. Using (A1) we substitute $\bar{x}$ to (A12). Assuming slow rotation, $\Omega \rho = \bar{v}\ll 1$, we expand to the linear order in $\bar{v}$,

A13 $$\begin{aligned} \cos (\Omega \bar{t}+\psi )&=\cos \varphi +V\bar{v}\,\sin ^2\varphi +O(\bar{v}^2), \end{aligned}$$

A14 $$\begin{aligned} \sin (\Omega \bar{t}+\psi )&=\sin \varphi -V\bar{v}\,\sin \varphi \cos \varphi +O(\bar{v}^2). \end{aligned}$$

Integrations over $\psi$ and $\varphi$ are equivalent on $[0,2\pi )$. We find that $S^{x 0}\sim \int _0^{2\pi }d\varphi \,\sin \varphi = 0$ and

A15 $$\begin{aligned} S^{z 0}&\simeq -\mu \rho ^2\bar{\gamma }\gamma _{\tiny {\text{ V }}}\int \limits _0^{2\pi }d\varphi \, (V\bar{v} +\cos \varphi ) \nonumber \\&=-2\pi \mu \rho ^2\bar{\gamma }\gamma _{\tiny {\text{ V }}}V\bar{v} =-\gamma _V V\bar{S}. \end{aligned}$$

Similarly we calculate the energy,

A16 $$\begin{aligned} \begin{aligned} P^{0}&=\mu \rho \int \limits _0^{2\pi } d\psi \, u^{0}= \mu \rho \int \limits _0^{2\pi } d\psi \,\gamma _{\tiny {\text{ V }}}(\bar{u}^0+V\bar{u}^x)\\&\simeq \mu \rho \bar{\gamma }\gamma _{\tiny {\text{ V }}}\int \limits _0^{2\pi } d\psi \,(1+V\bar{v}\cos \varphi )=2\pi \mu \rho \bar{\gamma }\gamma _{\tiny {\text{ V }}}=\gamma _V M. \end{aligned}\end{aligned}$$

Here $\bar{S}$ and *M* are given by (A4) and (A5). The CI shift (4.9) of the rotating ring becomes

A17 $$\begin{aligned} \Delta R^{x,y,0}=0 \quad \Delta R^{z}=-V{\bar{S}\over M}. \end{aligned}$$

We emphasize that this relativistic derivation holds for all speeds $-1<V< 1$; only $\bar{v}$ is assumed to be small. Equation (A17) is the relativistic generalization of Muller’s [6] and Rębilas’s [7] results. Because $\Delta R^{z}$ in (A17) is linear in the spin, it does not depend on the distribution of the rotating matter. It applies to any axisymmetric gyroscope, not only a ring.

## Appendix B: Motion of the center of mass

Here we focus on the influence of the classical spin on the orbit of CM. Treating spin as a perturbation, we characterize the motion of the CM in the direction perpendicular to the zeroth order orbital plane. We neglect radiation effects, proportional to the square of the particle’s charge, and the magnetic moment. The linear momentum obeys [5]

B1 $$\begin{aligned} {d\over d\tau }\Big (MU^{\alpha }+S^{\alpha \beta }w_{\beta }\Big )&=f^{\alpha }, \end{aligned}$$

B2 $$\begin{aligned} f^{\alpha }&=qF^{\alpha \beta }U_{\beta }. \end{aligned}$$

The spin affects the motion via the second term in (B1). Of higher order in derivatives, it may cause unphysical instabilities similar to self-acceleration due to radiation reaction. To avoid spurious solutions, we treat higher derivatives perturbatively. We consider the spin to orbital angular momentum ratio as small, move the spin-dependent term to the right hand side, and interpret it as an extra force $\Delta f$,

B3 $$\begin{aligned} \Delta f^{\alpha }=-{d\over d\tau }\Big (S^{\alpha \beta }w_{\beta }\Big ) =-w_{\beta }{d \over d\tau }S^{\alpha \beta } -S^{\alpha \beta }{d \over d\tau }w_{\beta }. \end{aligned}$$

Because of (4.5), antisymmetry of $S^{\alpha \beta }$, and the orthogonality property $w^{\alpha }U_{\alpha }=0,$ the first term vanishes. For an electromagnetic interaction (B2), Eq. (B1) becomes

B4 $$\begin{aligned} M w^{\alpha } = q F^{\alpha \beta }U_{\beta }-S^{\alpha \beta }{d \over d\tau }w_{\beta } \end{aligned}$$

We look for solutions in the form of a series

B5 $$\begin{aligned} w^{\alpha }=w^{\alpha }_{(0)}+w^{\alpha }_{(1)}+\cdots , \end{aligned}$$

and similarly for *R* and *S*. Note that our goal is to find the effect of the spin; we compare the motion with that of a spinless particle of the same energy. Thus we assume that the 4-velocity *U* is not perturbed. In the first two orders, Eqs. (B1, B4) reduce to

B6 $$\begin{aligned} M w^{\alpha }_{(0)}= &   q F^{\alpha \beta }U_{\beta }, \end{aligned}$$

B7 $$\begin{aligned} M w^{\alpha }_{(1)}= &   - S^{\alpha \beta }_{(0)}{d \over d\tau }w_{(0)\,\beta }. \end{aligned}$$

The zeroth order (B6) is the same as for a spinless particle, with circular orbits in static external Maxwell fields. To compare orbits of particles with and without spin, we assume that the kinetic energy in the Lab frame and the external field are the same in both cases. The velocity *V* and the $\gamma$-factor are therefore treated as fixed.

The first order equation (B7) takes the form

B8 $$\begin{aligned} Mw^\alpha _{(1)} = - {q\over M}S^{\alpha \beta }_{(0)}F_{\beta \lambda }w_{(0)}^\lambda . \end{aligned}$$

The only non-zero components of the nucleus’ field are $F^{i0}$. Equation (4.9) relates $S^{\alpha 0}$ to the CI shift $\Delta R ^{\alpha }$,

B9 $$\begin{aligned} M w^{i}_{(0)}&= qU^0F^{0i}, \end{aligned}$$

B10 $$\begin{aligned} -{q\over M}S^{\alpha 0}_{(0)}F_{0i}w_{(0)}^{i}&= {1\over U^0}S^{\alpha 0}_{(0)}w_{(0)}^2 = M w_{(0)}^2\Delta R_{(0)}^{\alpha }. \end{aligned}$$

Equations (B7, B8) become

B11 $$\begin{aligned} w^{\alpha }_{(1)} = w_{(0)}^2\Delta R_{(0)}^{\alpha }. \end{aligned}$$

Thus the first order correction to the CM orbit is described by (B8). Because the external field has vanishing *z* component $F^{z\beta }=0$ the dynamics in *z* direction decouples from that in the orbital plane and with the same accuracy reduces to $w^{z}_{(1)} = w^2\Delta R^{z}$. Substituting

B12 $$\begin{aligned} \Delta R^{z}(t)={\tilde{S} V\over M} \sin \gamma \omega t \end{aligned}$$

from (5.8), with $w^2=\gamma ^4 \omega ^4 r^2$, leads to

B13 $$\begin{aligned} {d^2 z\over dt^2}=\gamma ^2\omega ^4 r^2{\tilde{S}V\over M} \sin \gamma \omega t. \end{aligned}$$

CM deviates from the zeroth-order orbital plane by

B14 $$\begin{aligned} z=-\omega ^2 r^2\,{\tilde{S}V\over M}\sin \gamma \omega t=-V^2\Delta R^{z}(t). \end{aligned}$$

## Code Availability Statement

My manuscript has no associated code/software. [Author’s comment: Code/Software sharing not applicable to this article as no code/software was generated or analysed during the current study.]
